# IRES-mediated translation of foot-and-mouth disease virus (FMDV) in cultured cells derived from FMDV-susceptible and -insusceptible animals

**DOI:** 10.1186/s12917-016-0694-8

**Published:** 2016-03-31

**Authors:** Takehiro Kanda, Makoto Ozawa, Kyoko Tsukiyama-Kohara

**Affiliations:** Department of Animal Hygiene, Joint Facility of Veterinary Medicine, Kagoshima University, Kagoshima, Kagoshima Japan; Transboundary Animal Disease Center, Joint Facility of Veterinary Medicine, Kagoshima University, Kagoshima, Kagoshima Japan

**Keywords:** FMDV, IRES, ITAF, ITAF_45_, 4E-BP1, PTB, Translation

## Abstract

**Background:**

Foot-and-mouth disease virus (FMDV) possess a positive sense, single stranded RNA genome. Internal ribosomal entry site (IRES) element exists within its 5′ untranslated region (5′UTR) of the viral RNA. Translation of the viral RNA is initiated by internal entry of the 40S ribosome within the IRES element. This process is facilitated by cellular factors known as IRES trans-acting factors (ITAFs).

Foot-and-mouth disease (FMD) is host-restricted disease for cloven-hoofed animals such as cattle and pigs, but the factors determining the host range have not been identified yet. Although, ITAFs are known to promote IRES-mediated translation, these findings were confirmed only in cells derived from FMDV-insusceptible animals so far.

We evaluated and compared the IRES-mediated translation activities among cell lines derived from four different animal species using bicistronic luciferase reporter plasmid, which possesses an FMDV-IRES element between *Renilla* and *Firefly* luciferase genes. Furthermore, we analyzed the effect of the cellular factors on IRES-mediated translation by silencing the cellular factors using siRNA in both FMDV-susceptible and -insusceptible animal cells.

**Results:**

Our data indicated that IRES-mediated translational activity was not linked to FMDV host range. ITAF_45_ promoted IRES-mediated translation in all cell lines, and the effects of poly-pyrimidine tract binding protein (PTB) and eukaryotic initiation factor 4E-binding protein 1 (4E-BP1) were observed only in FMDV-susceptible cells. Thus, PTB and 4E-BP1 may influence the host range of FMDV.

**Conclusions:**

IRES-mediated translation activity of FMDV was not predictive of its host range. ITAF_45_ promoted IRES-mediated translation in all cells, and the effects of PTB and 4E-BP1 were observed only in FMDV-susceptible cells.

## Background

Foot-and-mouth disease (FMD) is a highly contagious infectious disease in cloven-hoofed animals such as cattle, pigs, and other related species [1]. It is considered an endemic in several countries in Asia, Africa, and South America. Typical clinical signs of FMD include vesicle formation, and erosion of cutaneous mucosae and hairless parts of the skin proximal to the mouth and the hoofs. While FMD is rarely lethal in adult animals, it can induce myocarditis in newborn animals, which leads to high mortality [2]. In countries with endemic FMD, FMD-related mortality of young animals and decreased productivity (reduced milk production and animal weight loss) in adult animals can lead to major economic losses. In industrialized countries that are normally free of FMD, the costs associated with the control and eradication of an outbreak can reach several billion US dollars, which also include indirect losses due to trade restrictions on animal products [3].

FMDV possesses a positive sense, single stranded RNA as its viral genome and belongs to the *Aphtovirus* genus of the *Picornaviridae* family. FMDV possesses an internal ribosomal entry site (IRES) element within the 5′ untranslated region (5′UTR), and virus proteins are synthesized by IRES-mediated translation [1, 4]. It is known that, like FMDV, poliovirus (PV) and encephalomyocarditis virus (EMCV) belonging to the *Picornaviridae* family, and hepatitis C virus (HCV) belonging to the *Flaviviridae* family, possesses a virus-specific IRES element within the 5′UTR, and virus proteins are synthesized by IRES-mediated translation [5, 6]. According to the RNA secondary structure, picornavirus IRESs can be classified into five types designated I (PV), II (FMDV), III (hepatitis A virus), IV (HCV-like), and V (aichivirus-like) [7]. Although FMD primarily affects cloven-hoofed animals such as cattle and pigs [1], the factors that determine the host range of FMDV have not yet been identified. Usually, eukaryotic mRNA is translated by cap-dependent translation, which is initiated by recognition of the cap structure at the 5′ end of the mRNA by the 43S ribosome [8]. Virus mRNA with a short 5′UTR (<100 nucleotides) containing no AUG can facilitate protein synthesis in a cap-dependent manner, similar to most types of eukaryotic mRNAs [5, 6]. Cap-independent translation is mediated by the IRES [5, 6] and involves 3′-UTR cap-independent translation enhancer (3′-CITE)-mediated initiation [9, 10]. Vpg interacts with the cap-binding protein eIF4E to modulate translation [11, 12]. The translation of eukaryotic mRNA is halted or significantly suppressed by cleavage of eIF4G with picornavirus protease (e.g., PV 2A^pro^ and FMDV L^pro^), whereas protein synthesis directed by PV or EMCV-IRES is stimulated [13, 14]. FMDV L^pro^ can enhance translation driven by all picornavirus IRESs, even after inactivation of eIF2 by phosphorylation [15].

The FMDV-IRES element contains five domains, and each of these domains forms a specific three-dimensional conformation to directly bind to the 40S ribosome and initiate IRES-mediated translation [16, 17]. In addition to canonical eukaryotic initiation factors (eIFs), which are essential to initiate cap-dependent translation, IRES transacting factors (ITAFs), which specifically bind to the individual domains of the IRES element and stabilize its three-dimensional structure, are required to facilitate IRES-mediated translation [18, 19]. In previous reports, it was revealed that ITAF_45_ and polypyrimidin tract binding protein (PTB) plays an important role in facilitating IRES-mediated translation of FMDV [20–22].

On the other hand, eukaryotic initiation factor 4E (eIF4E), a cap-binding protein, is an essential cellular factor that initiates cap-dependent translation; however, some viral mRNAs with IRESs can escape the eIF4E regulatory pathway [23]. The translation inhibitor, eIF4E-binding protein 1 (4E-BP1), binds eIF4E in its dephosphorylated form and is phosphorylated by stimulation with insulin or epidermal growth factors to dissociate from eIF4E [24, 25] after phosphorylation by mammalian target of rapamycin complex 1 (mTORC1) [26]. The free-eIF4E can promote cap-dependent translation by forming eIF4F with other eIFs [27]. However, once 4E-BP1 is dephosphorylated due to stress, it binds to eIF4E tightly, and cap-dependent translation is suppressed because eIF4F cannot be formed [28, 29]. EMCV and PV have been reported to dephosphorylate 4E-BP1, which may block host protein synthesis [29]. Because eIF4E is not essential for some types of virus IRES-mediated translation, it is possible that dephosphorylation of 4E-BP1 could facilitate IRES-mediated translation of FMDV [29–31].

However, these results were obtained in an experiment using cell lines derived from mice or hamsters, which are not the host animals of FMDV. Hence, whether these findings are also observed in cells derived from the FMDV host animals have not yet been analyzed.

Clove-hoofed animals are the main FMDV hosts, but the virus or cellular factors that determine the host range have not yet been identified. In this study, to analyze whether the host range of FMDV is determined by its IRES-mediated translation or not, we evaluated and compared IRES-mediated translation activities among the cell lines derived from different animal species, including both host and non-host animals (host animals: bovine and swine, non-host animals: human and canine). Furthermore, to confirm whether previous findings about ITAFs and 4E-BP1 were also observed in the host animal cells of FMDV, we analyzed the effect of the ITAFs and 4E-BP1 for IRES-mediated translation of FMDV in both host and non-host animal cells.

## Results

### Evaluation and comparison of IRES-mediated translation activity of FMVD in various animal cell lines

**Fig. 1:**
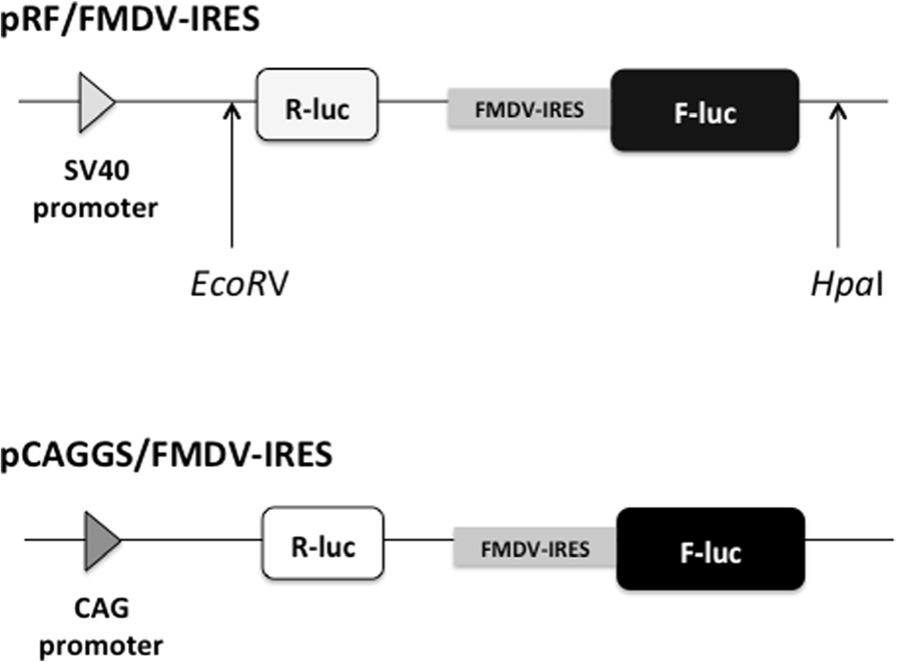
pRF/FMDV-IRES and pCAGGS/FMDV-IRES plasmid construction. Structure of the bicistronic luciferase reporter construct containing the FMDV-IRES element located between *Renilla* luciferase and firefly luciferase (pRF-FMDV-IRS). Reporter gene was excised from this plasmid construct using the restriction enzymes *Eco*RV and *Hpa*I, and was o the pCAGGS/MCS(F) vector treated with *Sma*I and rAPid Alkaline Phosphatase using Mighty Mix

To evaluate IRES-mediated translational activity, we constructed a bicistronic reporter plasmid [30]. *Eco*RV and *Hpa*I were used to excise the reporter gene from pRF/FMDV-IRES, which has an FMDV-IRES element between *Renilla* luciferase and firefly luciferase genes. The extracted reporter gene was then inserted into the pCAGGS/MCS(F) vector, as the CAG promoter is functional in most cell types [32]. This plasmid construct was named pCAGGS/FMDV-IRES (Fig. 1).

**Fig. 2:**
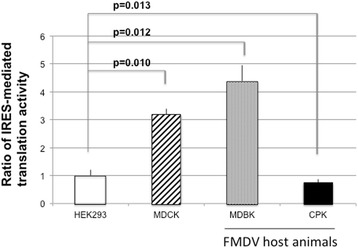
Evaluation of IRES-mediated translation activity. HEK293, MDCK, MDBK, and CPK cells were transfected with pCAGGS/FMDV-IRES. 24 h following transfection, *Renilla* luciferase and firefly luciferase units were measured using the Dual-Luciferase Reporter Assay System, and the ratio of IRES-mediated translation activity to that in HEK293 cells was evaluated. Experiments were performed in triplicates and error bars indicate S.D. Student’s *t*-tests were performed to calculate *p* values

This plasmid was then transfected into cell lines derived from the kidney epithelium of different animal species (HEK293 cells: human, MDCK cells: canine, MDBK cells: bovine, and CPK cells: swine). Amongst the assessed animal species, bovine and swine cell lines were derived from animals that are the natural hosts for FMDV. The bicistronic mRNA that contains the FMDV-IRES element between *Renilla* luciferase and firefly luciferase genes was produced in the transfected cells. 24 h following transfection, both *Renilla* luciferase and firefly luciferase activities in the cells were serially measured. *Renilla* luciferase activity represented cap-dependent translation, and firefly luciferase activity represented IRES-mediated translation. To evaluate IRES-mediated translational activity, the ratio of IRES-mediated translation to cap-dependent translation was calculated (Fig. 2). It was assumed that IRES-mediated translational activity of FMDV would be much higher in the cell lines derived from host animals of FMDV compared with cells derived from animals that are not natural hosts of FMDV. Previous studies have shown that IRES-mediated translational activity in CPK cells was higher than in MDBK cells, which may be due to efficient FMDV replication in swine cells [33]. In this study, although differences in IRES-mediated translational activity were observed among the cell lines, the translation activity of FMDV in FMDV-susceptible-host-derived cells was comparable to that of FMDV-insusceptible-host-derived cells. Furthermore, IRES-mediated translational activity in CPK cells was lower than that in MDCK cells, which were derived from canines (non-host animal of FMDV). Therefore, IRES-mediated translation activity cannot be used to accurately determine the host range of FMDV.

### The effects of cellular factors on IRES-mediated translation of FMDV

The cellular factors ITAF and eIF play important roles in facilitating IRES-mediated translation [20–22]. However, these findings were obtained by using cell lines derived from FMDV-insusceptible animals.

**Fig. 3:**
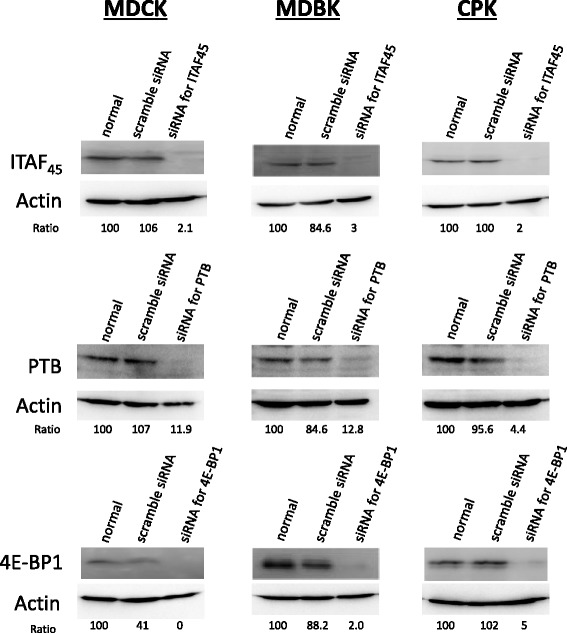
The effect of siRNAs on the cellular factors. The siRNAs targeting ITAF45, PTB, and 4E-BP1 were reverse transfected into MDCK, MDBK, and CPK cells using Lipofectamine RNAiMAX, and incubated for 48 h. After 48-h treatment, western blot analysis was performed on cell lysates to confirm the effect of siRNAs. Protein bands were quantified using an LAS1000UVmini. The protein intensity was divided by that of actin, and the normalized ratio (100 %) was indicated

**Fig. 4:**
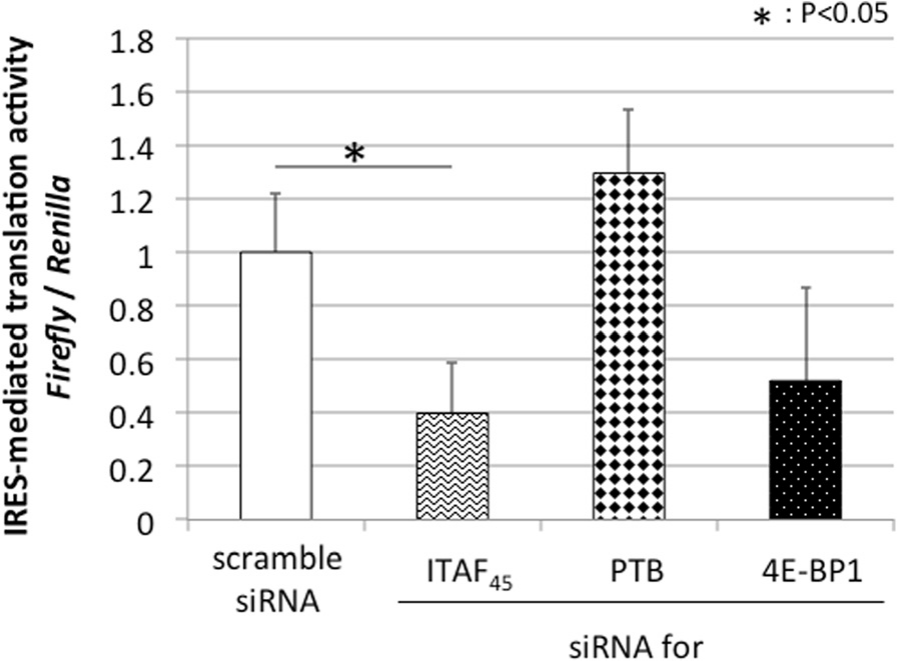
The effect of siRNAs on IRES-mediated translation in MDCK cells. After 48 h of siRNA treatment, cells were further transfected with plasmid construct. 24 h following transfection, *Renilla* luciferase and firefly luciferase units were measured using the Dual-Luciferase Reporter Assay System and IRES-mediated translation activity in MDCK cells. Experiments were performed in triplicates and error bars indicate S.D

**Fig. 5:**
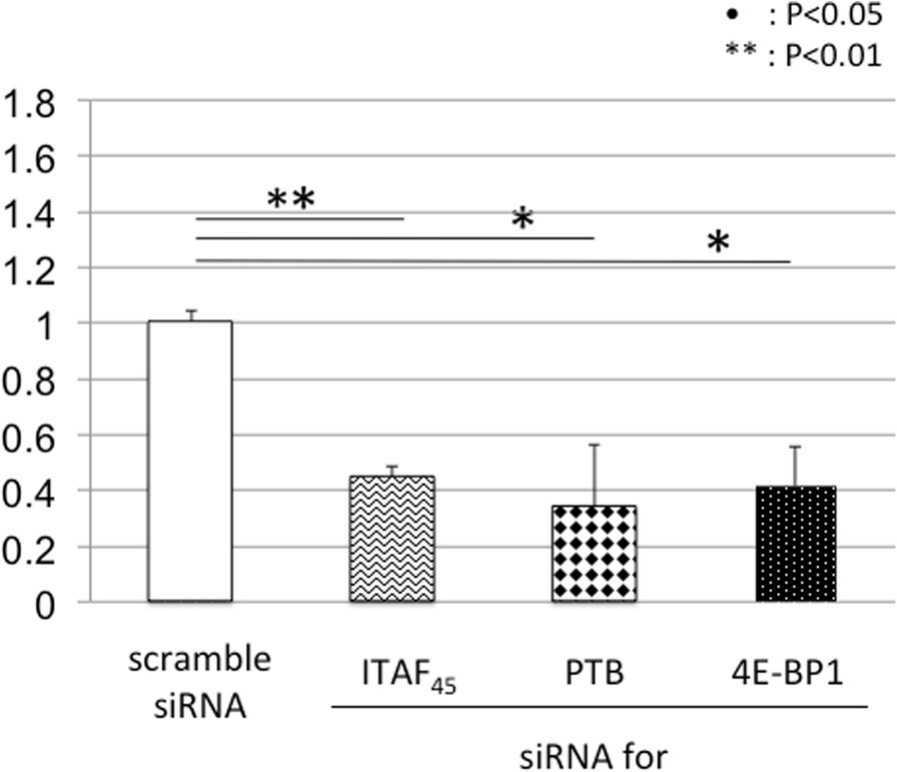
The effect of siRNAs on IRES-mediated translation in MDBK cells. After 48 h of siRNA treatment, cells were further transfected with plasmid construct. 24 h following transfection, *Renilla* luciferase and firefly luciferase units were measured using the Dual-Luciferase Reporter Assay System and IRES-mediated translation activity in MDBK cells. Experiments were performed in triplicates and error bars indicate S.D

**Fig. 6:**
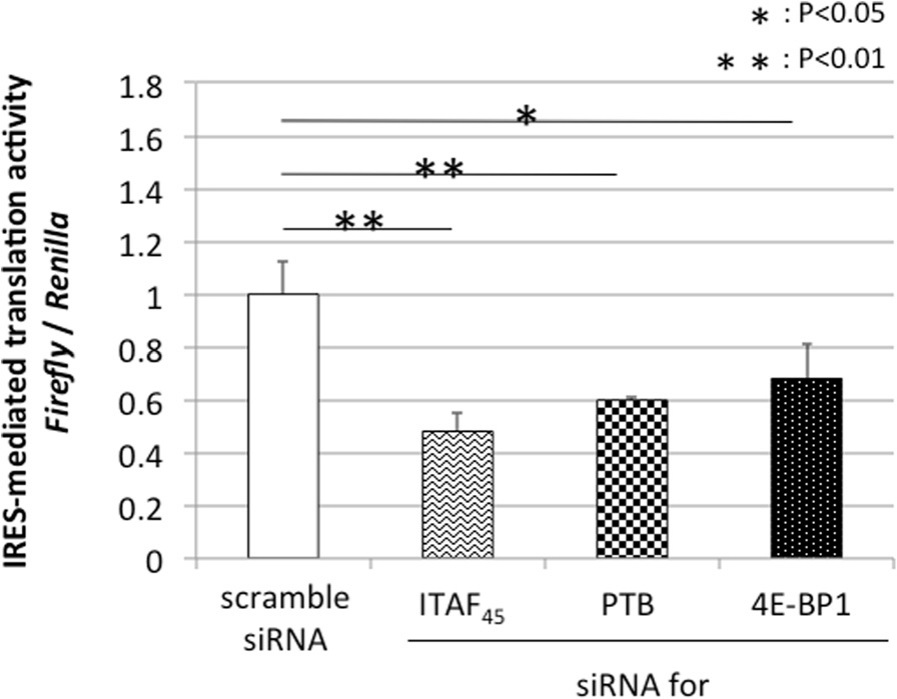
The effect of siRNAs on IRES-mediated translation in CPK cells. After 48 h of siRNA treatment, cells were further transfected with plasmid construct. 24 h following transfection, *Renilla* luciferase and firefly luciferase units were measured using the Dual-Luciferase Reporter Assay System and IRES-mediated translation activity in CPK cells. Experiments were performed in triplicates and error bars indicate S.D

Therefore, to analyze the effect of ITAFs and 4E-BP1, the expression of ITAF_45_, PTB, and 4E-BP1 was inhibited using siRNAs. The effect of siRNA was assessed by western blot analysis (Fig. 3). Following siRNA treatment, cells were further transfected with the reporter plasmid and evaluated for IRES-mediated translational activity (Figs. 4, 5 and 6). We found that inhibition of ITAF_45_ expression suppressed IRES-mediated translational activity in cell lines derived from both FMDV-susceptible and FMDV-insusceptible animals. Inhibition of PTB and 4E-BP1 reduced IRES-mediated translational activity only in the FMDV-susceptible host cell lines (MDBK *p* < 0.05, and CPK *p* < 0.01). Our results show silencing of ITAF_45_, PTB and 4E-BP1 in CPK cells can significantly suppress IRES-mediated translational activity.

## Discussion

In this study, we evaluated IRES-mediated translational activity in HEK293, MDCK, MDBK, and CPK cells to determine whether this process differs between cells derived from FMDV-susceptible and FMDV-insusceptible animals. Although difference in IRES-mediated translational activity was observed among these cell lines, it was not predictive of FMDV host range. Thus, other cellular processes/interactions that are distinct from IRES-mediated translation are needed when determining the host range of FMDV. For example, it was recently reported that the amino acid sequence of integrin protein, which is a candidate FMDV receptor, is different between host and non-host animals [34]. In the host animals, there is also a difference in the expression of integrin proteins between FMDV-susceptible organs and FMDV-insusceptible organs [35]. Furthermore, diverse innate immune reactions have been shown to be initiated against FMDV infection in different animal species, including the host animals of FMDV [33]. The data obtained in this study indicate that IRES-mediated translation cannot be used to accurately define FMDV host range.

Next, we analyzed the effect of ITAF_45_, PTB, and 4E-BP1 on IRES-mediated translation of FMDV in cell lines derived from FMDV-susceptible and FMDV-insusceptible animals. Inhibition of ITAF_45_ expression suppressed IRES-mediated translation activity in all cell lines. However, silencing of PTB and 4E-BP1 suppressed IRES-mediated translation activity only in the cell lines derived from FMDV host animals. The active subunits of some cellular factors, including PTB, are generated by proteolysis, which has been correlated to FMDV-induced cytopathic effects [36]. Our findings from this study indicate that PTB, along with some other unidentified factors may be involved in facilitating IRES-mediated translation in FMDV-susceptible animals. The role of ITAFs in IRES-mediated translation has not yet been thoroughly characterized. Additional studies are required to determine the role of ITAFs in regulating FMDV-susceptibility of cells through IRES-mediated translation.

The inhibition of 4E-BP1 expression suppressed IRES-mediated translation activity in MDCK, MDBK, and CPK cells. These unexpected results suggest that IRES-mediated translation is dependent on 4E-BP1 (indicated by decrease in firefly luciferase activity, data not shown). Overexpression of 4E-BP1 has been previously shown to promote IRES-mediated translation of EMCV, and eIF4E availability has been shown to regulate IRES-mediated translation efficiency [37]. Although the translation of some viruses, such as poliovirus and vesicular stomatitis virus, has been shown to be promoted by phosphorylation of 4E-BP1, the translation of other viruses, such as adenovirus, has been shown to be promoted by dephosphorylation of 4E-BP1 [33, 38]. Furthermore, 4E-BP1 was previously shown to be involved in other cell activities such as regulation of cell growth and oncogenesis [39, 40]. Together with previous findings, our study indicates that 4E-BP1 regulates IRES-mediated translation of FMDV through complex mechanisms, and may play differential roles among various animal species. Further studies are required to characterize these potential novel functions of 4E-BP1.

## Conclusions

IRES-mediated translation activity of FMDV was not predictive of its host range. ITAF_45_ promoted IRES-mediated translation in all cells, and the effects of PTB and 4E-BP1 were observed only in FMDV-susceptible cells. Eukaryotic initiation factor 4E-BP1 unexpectedly interacted with IRES-mediated translation in cells.

## Methods

### Cell lines and culture

The human kidney cell line (HEK293) and bovine kidney cell line (MDBK) used in this study were obtained from the American Type Culture Collection (ATCC), and were maintained in Dulbecco’s modified Eagle’s medium (DMEM; Nissui) supplemented with 10 % fetal calf serum (FCS; Bovogen). The canine kidney cell line (MDCK) was originally from ATCC and was maintained in Eagle’s minimum essential medium (MEM; Life Technologies) supplemented with 5 % newborn calf serum (NCS; Hazleton). The porcine kidney cell line (CPK) [41] was maintained in MEM supplemented with 10 % FCS. All cell lines were cultured at 37 °C in 5 % CO_2_.

### Plasmids

The pRF/FMDV-IRES plasmids were kindly provided to us by Dr. Hirasawa (Memorial University of Newfoundland). Reporter genes were excised from pRF/FMDV-IRES using the restriction endonucleases *Eco*RV (Toyobo) and *Hpa*I (NEB). pCAGGS/FMDV-IRES was generated by inserting a reporter gene into pCAGGS/MSC(F), which was then treated with SmaI (Takara) and rAPid Alkaline Phosphatase (Roche) using Mighty Mix (Takara). DNA fragments were purified with the Big Dye XTerminator Purification kit, followed by sequencing via capillary electrophoresis on the ABI PRISM310 genetic analyzer.

### Short interfering RNA assay

Short interfering RNAs (siRNAs) targeting human-ITAF_45_ were used for MDCK, and MDBK cells. The siRNAs targeting mouse-ITAF_45_ were used for CPK cells. Both types of ITAF_45_-targeting siRNAs were composed of a mixture containing 3 siRNAs with 19–25 nucleotides (Santa Cruz Biotechnology). The siRNAs targeting human-PTB and human-4E-BP1 were as follows: 5′-GGCAGGAAATTCTGTATTG-3′, and 5′-GAGTCACAGTTTGAGATGGACATTTAA-3′, respectively (Life Technologies).

### Transfection

Cells (10^5^/well) were grown in 24-well plates with culture medium for 24 h to achieve 50–70 % confluency for transfection. Plasmid transfection was performed using Lipofectamine 2000 reagent (Invitrogen) according to manufacturer’s specification. The siRNA reverse transfection was performed using Lipofectamine RNAiMIX reagent (Invitrogen) according to manufacturer’s specification.

### Luciferase assay

Cells were washed once with PBS (−), and lysed with passive lysis buffer (Promega). *Renilla* luciferase and firefly luciferase activities were measured using the Dual-Luciferase Reporter Assay System (Promega). Cell lysates (10 μL) were mixed with a 10-fold diluted reagent (100 μL), and luminescence was measured with the GloMax 96 Microplate for 10 s. Luciferase activity was quantified as the relative fluorescent intensity in the 10-s interval.

### Western blot analysis

Cells were washed twice with PBS, and lysed with RIPA buffer containing 0.1 % SDS, 10 μg aprotinin/mL, 100 μg PMSF/mL and 1 % phosphatase inhibitor cocktail (Sigma). Cell lysates were subjected to SDS-PAGE, and the resolved proteins were transferred to a PVDF membrane (Millipore). The membrane was blocked with 5 % Block Ace (Yukijirushi) in Tris buffered saline (TBS) containing 0.1 % Tween-20, and was probed with the following primary antibodies: anti-ITAF45 (Santa Cruz Biotechnology), anti-PTB (Cell Signaling), anti-4E-BP1 (Cell Signaling), and anti-βactin (SIGMA)]. The membranes were then washed 3 times with 0.05 % Tween- 20-TBS for 10 min. Secondary antibodies (peroxidase-conjugated anti-rabbit or anti-mouse IgG; DAKO) were subsequently added, and specific protein bands were visualized with enhanced chemical luminescence (GE Healthcare).

### Statistical analysis

All data are presented as means ± SEM from three independent experiments. Statistical analysis was performed using Student’s *t*-test to evaluate significant differences (*P < 0.05, **P < 0.01).

## Ethical approval and consent to participate

All experiments were approved by the ethics committee of Kagoshima University. This study did not include animal experiments.

## Consent for publication

Not applicable.

## Availability of data and materials

Data and materials are available upon request.

## Abbreviations

FMDV: Foot-and-mouth disease virus
IRES: Internal ribosomal entry site
5′UTR: 5′ untranslated region
ITAFs: IRES trans-acting factors
PTB: Poly-pyrimidine tract binding protein
4E-BP1: 4E-binding protein1
